# Interactions between wild pigs and the spread of disease

**DOI:** 10.7554/eLife.105293

**Published:** 2024-12-18

**Authors:** Mercury Shitindo

**Affiliations:** 1 Africa Bioethics Network Nairobi Kenya; 2 University of Zaragoza Zaragoza Spain

**Keywords:** wild pigs, disease, transmission, ecology, crops, biodiversity, Other

## Abstract

Tracking wild pigs with GPS devices reveals how their social interactions could influence the spread of disease, offering new strategies for protecting agriculture, wildlife, and human health.

**Related research article** Proboste T, Turnlund A, Bengsen A, Gentle M, Wilson C, Harriott L, Fuller RA, Marshall D, Magalhães RJS. 2024. Quantifying feral pig interactions to inform disease transmission networks. *eLife* **13**:RP102643. doi: 10.7554/eLife.102643.

Across landscapes worldwide, wild pigs are more than a nuisance – they are a global economic and ecological catastrophe. They pose threats to agriculture by destroying crops, to biodiversity by competing with native species, and to humans and farm animals by spreading disease. In the United States alone, wild pigs cause an estimated $120 billion in damages every year (Risch et al., 2021) and in Australia it has been estimated that a single outbreak of foot-and-mouth disease could cost the economy around $50 billion (Buetre, 2013).

To tackle the threat of disease outbreaks, it is important to understand how diseases spread through populations of wild (or feral) pigs, so it necessary to know how often wild pigs come into contact with each other. However, it is challenging to measure such contact rates for wild pigs because they are highly social and because they roam freely across vast landscapes. Now, in eLife, Tatiana Proboste (University of Queensland) and colleagues at Queensland and other research institutes in Australia report a new approach to collecting such data (Proboste et al., 2024).

Using GPS collars to track 146 wild pigs across diverse terrain in eastern Australia over six years, the team uncovered intricate patterns of animal movement and interaction. Their findings revealed crucial insights into how these animals socialize and move through their territories, offering new ways to predict and control the spread of dangerous diseases like foot-and-mouth disease, African swine fever, and zoonotic infections that can spread to humans.

The experiments showed that wild pigs organize themselves into distinct social groups, or "sounders", typically made up of adult females and their young. Adult males, in contrast, lead more solitary lives (Spencer et al., 2005). Using GPS data, Proboste et al. found that interactions between the pigs within sounders were frequent and cohesive, while interactions between different groups were relatively rare and mediated by roaming males. This dynamic is crucial for understanding disease transmission. Diseases are likely to spread quickly within a single sounder due to high levels of contact, but solitary males that move between groups create a potential pathway for diseases to spread more broadly. These findings echo patterns observed in wild boar populations in Europe, where males act as ‘bridges’ between otherwise isolated groups (Podgórski et al., 2018).

Conventional strategies for controlling disease outbreaks often focus on culling adult females to curb population growth (Bengsen et al., 2014). However, the results of Proboste et al. suggest a shift in strategy: culling adult males might be more effective because it could prevent the disease spreading from group to group. The researchers also uncovered seasonal variations in pig interactions, with contact rates peaking in summer – information that can be used to ensure that disease control measures are implemented when they are most likely to be effective. The inclusion of real-word data about wild pigs in models of disease transmission, such as the Australia Animal Disease Spread model (Bradhurst et al., 2015), will help the relevant authorities to respond to disease outbreaks more effectively.

As the global threat of diseases like African swine fever continues to grow, understanding the social networks of wild pigs has never been more important. The study by Proboste et al. highlights the power of combining technology and ecological insights to address complex challenges in public and animal health. While this study marks a significant step forward, questions remain.

How do environmental factors influence the seasonal patterns of pig interactions that were observed? Could machine learning help predict when and where disease transmission is most likely based on pig movement data? Answering these questions will require interdisciplinary approaches that combine ecology, epidemiology and advanced data analytics. By understanding how wild pigs interact and move across different habitats, we can develop more targeted and effective strategies to protect agriculture, biodiversity and human health from disease outbreaks.
